# Concerns of iodized salt and its effects for women seeking antenatal care in Pakistan

**DOI:** 10.12669/pjms.38.8.5220

**Published:** 2022

**Authors:** Faryal Shaikh, Seema N. Mumtaz, Aqeel Ahmed Shaikh

**Affiliations:** 1 Dr. Faryal Shaikh, MBBS, MPH. Senior Lecturer in Department of Community Medicine, Fazaia Ruth Pfau Medical College (FRPMC), Karachi, Pakistan; 2 Prof. Dr. Seema N Mumtaz, MBBS, M.Phil, MPH, MBA (Health) DCPS (HCSM). Chairperson and HOD of Community Health Sciences Department, Karachi Institute of Medical Sciences (KIMS) Malir Cantt, Karachi, Pakistan; 3 Prof. Dr. Aqeel Ahmed Shaikh, BDS, MCPS, FICCDE. Vice Principal and HOD Dentistry Department, Karachi Medical and Dental College (KMDC), Karachi, Pakistan

**Keywords:** Iodized Salt, Concerns and effects, Antenatal care, Pakistan

## Abstract

**Background and Objective:**

Pregnant women are the most susceptible group for Iodine deficiency disorder (IDD) whose neonate are at the risk of brain impairment, if they are iodine deficient in utero. The study was carried out to analyze the concerns and effects regarding iodized salt and IDD in women seeking antenatal care in Pakistan

**Methods:**

A descriptive cross-sectional study was conducted in Obstetrics OPD at Civil Hospital Karachi from April 2017 to January 2018. In this study, antenatal care seeking women (n=360) visiting obstetric outpatient department (OPD) at public sector tertiary care hospital of Karachi were interviewed face to face using a structured questionnaire. Systematic random sampling method was employed. Kruskal Wallis test was applied to assess the significance among study variables.

**Results:**

Sixty-three (63.6%) of pregnant women heard about iodized salt. Approximately 40.6% of them received iodized salt related information through mass media. Ninety (90.6%) were ignorant that their unborn child needs iodine for brain development. A statistically significant association was found between the educational status (p<0.001), household income (p<0.001), age (p=0.016), ethnicity (p=0.018), trimester (p=0.005) with the knowledge of study participants regarding iodized salt and IDD.

**Conclusion:**

There is an immense need to address the present concerns of women seeking antenatal care by advocacy and health education on individual and at mass level regarding the use of iodized salt among women seeking antenatal care. Advocacy can be done by governmental initiatives, medical personnel and through mass media in all tertiary care hospitals of Pakistan.

## INTRODUCTION

IDDs during pregnancy and infancy are the leading cause of a preventable cognitive impairment and intellectual disabilities which irreversibly hinders development of brain and growth in children.^1^ Iodine is a trace element essential for the synthesis of thyroid hormone by the thyroid gland. Pregnant women are the most susceptible group for IDD whose neonate are at the risk of brain impairment, if they are iodine deficient in utero. Globally about 241 million (29.8%) of school age children are estimated to have inadequate iodine intake. Among 241 million children, 76 million belong to the WHO South East Asia region and 58 million children belong to African region.^2^ IDD due to insufficient thyroid hormone production has numerous adverse effects on health.^3^

Globally two billion individuals have an inadequate iodine intake. The regions which are particularly affected with iodine deficiency are South Asia and Sub-Saharan Africa.^4^ Mild iodine deficiency is also affecting approximately 50% Continental Europe.^5^ According to National Nutrition Survey (NNS) of Pakistan, prevalence of low Urinary Iodine excretion among women of reproductive age (i.e., 17.5%) is higher in countryside as compared to metropolitan areas.^6^ In 1993, Universal Salt Iodization (USI) has been recommended as the main strategy to achieve elimination of IDD. It was introduced in Pakistan in 1994^7^ as a widely used cost effective strategy According to NNS, the reported consumption of iodized salt at household level in Pakistan is 79.6%^8^ which is less than the WHO benchmark of > 90% household coverage of iodized salt to achieve the goal of USI.^9^ Pakistan is moving forward towards attaining USI, the program focus has now been shifted for sustaining USI.^8^ Therefore, The study was carried out to analyze the concerns and effects regarding iodized salt and IDD in women seeking antenatal care in Pakistan.

## METHODS

A descriptive cross-sectional study was carried out in Obstetrics OPD at Civil Hospital Karachi. The study population included pregnant women aged 16-45 years seeking routine antenatal care and who were willing to participate in the study. Those who were having chronic diseases, taking chronic medications and who did not give consent to participate in the study were excluded. A Systematic random sampling method was employed in which every 5^th^ pregnant woman who were enrolled in the OPD register were included and interviewed after taking informed consent**.** Sample size was estimated using the software OpenEpi. Sample size estimation was based on proportion of inadequate iodine intake (37.4%) with 5% level of significance and 95% CI. The proportion of people with inadequate iodine intake for EMRO was taken from WHO global database i.e., 37.4%.^10^ Using the above mentioned values, the calculated sample size was found to be 360. Data was collected from April 2017 to January 2018. Pilot testing of a questionnaire was performed on 10% of respondents i.e., 36 pregnant women. After this final survey was implemented. Data collection tool included a validated structured questionnaire administered on 360 study participants through face to face interview.

There were two main sections in the questionnaire that included sociodemographic characteristics, assessment of level of knowledge of antenatal women regarding iodized salt. Study participants were asked regarding the iodized salt, sources of iodized salt related information, adverse effects of iodine deficiency and importance of iodized salt in pregnant women.

Data was entered and analyzed using SPSS version 22. Descriptive analysis was performed by calculating frequencies and percentages for categorical variables and mean and standard deviation for continuous variables. The Kolmogrov-Smirnov test was applied to determine the nature of response distribution. Association of Sociodemographic characteristics with the level of knowledge of antenatal women regarding the iodized salt and IDD were assessed through Kruskal Wallis test. Inferential statistics (Kruskal Wallis tests, p<0.05) were used to assess the significance among study variables. All analyses were performed using SPSS version 22.

Household income and education

1. Those who have an income of Rupees < 10,000 (USD < 48.88) or between Rupees 10,000-20,000 (USD=48.88-97.76) or are illiterate or able to read and write are considered in low socioeconomic status.
2. Those who have an income of Rupees 21,000-30,000 (USD=102.64-146.63) or have primary or secondary level of education are categorized in Middle Socioeconomic status.
3. Those who have an income of Rupees > 30,000 (USD > 146.63) or have higher level of education are considered in Upper Socioeconomic Status.

1 USD= 204.59 PKR on Monday, July 4^th^ ,2022

### Ethical consideration:

The study plan was approved by the Ethical Review Committee (Ref: of Baqai Medical University, Karachi, Pakistan. (16/2014/SI and Dated: February 8, 2016).

## RESULTS

Among 360 participants, (43.3%) belonged to the third trimester of pregnancy. Fifty percent of pregnant women belonged to the age group of 16-25 Years. A large percentage of study participants i.e., 46.1% (n=166/360) were found illiterate while 23.9% (n=86/360) had primary education and only 6.9% (n=25/360) achieved higher education. Around 87.2% of pregnant women belonged to low socioeconomic status. More than half of pregnant women 55% (n=198) had a monthly household income of Rs. 10,000-20,000 (Table-I).

**Table-I:** Demographic characteristics of the Antenatal care seeking women (n=360), Karachi, Pakistan.

Characteristic	Frequency (N)	Percentage (%)
***Age (years)***		
16-25	180	50.0
26-35	148	41.1
36-45	32	8.9
Mean Age	27 [±5.966]	
***Religion***		
Islam	355	98.6
Christian	3	0.8
Hindu	2	0.6
***Para***		
1-3	291	80.8
4-7	61	16.9
8-11	8	2.2
***Gravida***		
1-3	217	60.3
4-6	114	31.7
7-9	29	8.1
***Ethnic group***		
Punjabi	17	4.7
Saraiki	6	1.7
Muhajirs	116	32.3
Sindhi	52	14.4
Balochi	40	11.1
Pakhtoon	55	15.3
Others	77	20.6
***Family system***		
Nuclear	58	16.1
Combined	302	83.9
***Educational status***		
Illiterate	166	46.1
Able to read and write	22	6.1
Primary	86	23.9
Secondary	61	16.9
Higher education	25	6.9
***Trimester***		
1^st^	94	26.1
2^nd^	110	30.6
3^rd^	156	43.3
***Occupational status***		
House wife	346	96.1
Self employed	13	3.6
Employed	1	0.3
***Employment status of husband***		
Employed	338	93.9
Self employed	11	3.1
Unemployed	11	3.1
Monthly household income*		
Less than 10,000	99	27.5
10,000-20,000	198	55.0
21000-30000	52	14.4
More than 30,000	7	1.9
***Socio economic status***		
Upper	2	0.6
Middle	44	12.2
Low	314	87.2
Gestational week (Mean ± SD)	24.09 ± 11.18	

Missing data= 4.

Out of 360 participants, 82.5% (n=297) of respondents did not know about what iodine is. 63.6% (n=229/360) of the respondents heard about iodized salt. Among (n=229/360) participants who received iodized salt related information through mass media was 40.6%. About 90.8% (n=327/360) of respondents did not know about the consequences of iodine deficiency in newborn, children and adults (Table-II).

**Table-II:** Knowledge of antenatal care seeking women (n=360) regarding Iodized salt and IDD, Karachi, Pakistan.

Items in questionnaire	Frequency (N)	Percentage (%)
***Do you know what iodine is?***		
Mineral	50	13.9
Vitamin	4	1.1
Something in the food we eat	8	2.2
Other	1	0.3
Do not know	297	82.5
***Do you know about main sources of iodine?***		
Seafood	40	11.1
Vegetable	17	17
Meat	1	1
Dairy products	3	3
Other	4	4
Don’t know	295	81.9
***Have you ever heard about consequence of iodine deficiency?***		
Yes	46	12.8
No	314	87.2
***Can you enumerate any disease that results from iodine deficiency in our body?***		
Yes	48	13.3
No	312	86.7
***Have you ever heard about iodized salt?***		
Yes	229	63.6
No	131	36.4
***Sources of iodized salt-related information***		
Mass media	146	40.6
Print Media	4	1.1
Members of your family	35	9.7
Neighbors, acquaintances, friends	17	4.7
Heard from Doctor	9	2.5
From grocery store	9	2.5
Others	6	1.7
***Are you aware of the grave consequences of IDD in newborn, children & adult?***		
Abortion	3	0.8
Stillbirth	4	1.1
Congenital anomalies	5	1.4
Mental retardation	10	2.8
Perinatal mortality	0	0
Goitre	3	0.8
Heard about it first time	5	1.4
Don’t know	327	90.8
All	3	0.8
***Do you know that iodine deficiency in your children results in mental retardation?***		
Yes	36	10.0
No	324	90.0
***Do you know that during pregnancy your baby needs iodine for the brain development?***		
Yes	34	9.4
No	326	90.6
***Do you know iodine is required in which trimester in pregnancy?***		
1^st^ trimester	3	0.8
2^nd^ trimester	0	0
3^rd^ trimester	0	0
Do not know	357	99.2
***Does your health care provider inform you about importance and requirement of iodine durin***		
***g pregnancy?***		
Yes	8	2.2
No	352	97.8
***Do you know that iodine deficiency causes goiter?***		
Yes	32	8.9
No	328	91.1
***Do you know that iodized salt consumption is essential during pregnancy?***		
Yes	40	11.1
No	320	88.9

A statistically significant association was found between the educational status (p<0.001), household income (p<0.001), age (p=0.016, 0.004), religion (p=0.008), ethnicity (p=0.018) with the level of knowledge regarding iodine, iodized salt and IDD of antenatal care seeking women (Table-III).

**Table-III:** Association of level of knowledge towards Iodized salt, IDD with the demographic characteristics of antenatal care seeking women (n=360), Karachi, Pakistan.

Items in questionnaire	P-Value*							

	Age	Religion	Para	Gravida	Ethnicity	Education	Trimester	Income
Do you know what iodine is?	0.615	0.601	0.404	0.651	0.018	<0.001	0.168	<0.001
Do you know about main sources of iodine?	0.872	0.691	0.371	0.496	<0.001	<0.001	0.456	<0.001
Have you ever heard about consequence of iodine deficiency?	0.016	0.498	0.110	0.139	0.032	<0.001	0.600	<0.001
Can you enumerate any disease that results from deficiency of iodine in our body?	0.009	0.558	0.160	0.813	0.034	0.011	0.989	<0.001
Have you ever heard about iodized salt?	0.080	0.075	0.674	0.645	0.011	<0.001	0.424	<0.001
Sources of iodized salt-related information	0.295	0.206	0.151	0.802	0.324	0.496	0.842	0.309
Are you aware of the grave consequences of IDD in newborn, children & adult?	0.176	0.298	0.738	0.849	0.195	0.001	0.005	<0.001
Do you know that iodine deficiency in your children results in mental retardation?	0.070	0.367	0.128	0.311	0.011	0.007	0.418	<0.001
Do you know that during pregnancy your baby needs iodine for the brain development?	0.081	0.331	0.391	0.569	0.030	0.007	0.647	<0.001
Do you know iodine is required in which trimester in pregnancy?	0.115	0.979	0.733	0.412	0.768	0.005	0.216	0.897
Does your health care provider inform you about importance and requirement of iodine during pregnancy?	0.592	0.962	0.733	0.714	0.812	0.056	0.320	0.004
Do you know that iodine deficiency causes goiter?	0.065	0.299	0.060	0.397	0.009	0.009	0.331	<0.001
Do you know that iodized salt consumption is essential during pregnancy?	0.061	0.008	0.965	0.372	0.799	0.011	0.652	<0.001

Kruskal Wallis test.

## DISCUSSION

Our study findings revealed lack of concern about IDD, its consequences, benefits of iodine and importance of iodized salt in antenatal women. Ninety percent of respondents had no knowledge that iodine deficiency results in mental retardation and goitre. Despite the introduction of a National IDD Control Program in 1994, approximately half of population of Pakistan is afflicted with IDD. Since the guidelines of National USI program do not include the measures for the utilization of iodized salt in processed foods which is affecting program sustainability .However adequately iodized salt is not available and acceptable by several regions of community.^10^ Therefore the availability and fortification of iodine in processed foods may contribute for increasing iodine nutrition.^11^,^12^ The findings of present study are supported by studies conducted.^13^-^15^ In present study and Addis Ababa city, monthly household income and educational status of participants were associated with knowledge related to iodized salt.^15^ However, a study carried out in North West Ethiopia^16^ reported that 63.6% were unaware about iodized salt and 18% received information about iodized salt through mass media. Moreover, in another Ethiopian^17^ and South Indian^18^ study which reported that more than half of respondents were acquainted that iodine deficiency results in goiter. A major bulk of respondents heard about iodized salt.^17^,^18^ Only 16.6% respondents acquired information regarding importance of iodized salt through health worker. More than half of participants knew that iodine deficiency results in mental and growth retardation. The difference could be because of difference in educational status where half of respondent’s attained secondary education and 12.1% had acquired university education.^17^ In the present study the educational status of participants is quite low and 87.2% belong to low socio-economic status. In contrast to another study which was carried out in Bangladesh,^19^ a significant correlation was found between knowledge level and educational status (p < 0.05) of participants.^19^

In another study conducted in Somali Ethiopia^20^ which reported that most of the respondents were well aware about iodized salt and acknowledged mass media (31.3%) and health worker (8%) as a source of information. This difference could be because of their higher educational status.^20^ While in a study conducted in Norway^21^ which reported that 16.6% received information about iodine from their health professional, less than 17% of pregnant women replied that intake of iodine is important for normal fetal development. The difference could be due to difference in level of education, dietary habits and geographical location.^21^ The researcher through this study revealed lack of concern related to benefits of iodized salt and effects of IDD in antenatal women.

### Limitations of the study:

As this study carried out in a single tertiary care hospital, therefore the results cannot be generalized at population level. Limitation in retrieving information was observed among the study participants while conducting interview because of memory lapse.

## CONCLUSION

Pregnant women are the most vulnerable population which are lacking knowledge regarding the importance of iodized salt. There is an immense need to address the present concerns of women seeking antenatal care by advocacy and health education on individual and at mass level. Advocacy can be done by governmental initiatives, medical personnel and through mass media in all tertiary care hospitals of Pakistan regarding the use of iodized salt by women seeking antenatal care.

### Author’s Contribution:

**FS:** Conceived, designed, data analysis, interpretation, manuscript writing, and accountable for all aspects of the work related to accuracy and integrity of the work appropriately investigated and resolved.

**SM:** Reviewed, data analysis, interpretation and final approval of the version to be published.

**AS:** Reviewed, assisted in data analysis.
